# Predictors of Gastrointestinal Involvement in Children with IgA Vasculitis: Results from a Single-Center Cohort Observational Study

**DOI:** 10.3390/children11020215

**Published:** 2024-02-07

**Authors:** Donato Rigante, Cristina Guerriero, Sara Silvaroli, Filomena Valentina Paradiso, Giorgio Sodero, Francesco Laferrera, Francesco Franceschi, Marcello Candelli

**Affiliations:** 1 Department of Life Sciences and Public Health, Fondazione Policlinico Universitario A. Gemelli IRCCS, 00168 Rome, Italy; 2 Università Cattolica Sacro Cuore, 00168 Rome, Italy; 3 Department of Dermatology, Fondazione Policlinico Universitario A. Gemelli IRCCS, 00168 Rome, Italy; 4 Unit of Pediatric Surgery, Department of Woman and Child Health and Public Health, Fondazione Policlinico Universitario A. Gemelli IRCCS, 00168 Rome, Italy; 5 Department of Emergency Anesthesiological and Reanimation Sciences, Fondazione Policlinico Universitario A. Gemelli IRCCS, 00168 Rome, Italy; marcello.candelli@policlinicogemelli.it

**Keywords:** IgA vasculitis, Henoch-Schönlein purpura, 25-hydroxy-vitamin D, gastrointestinal complications, child, personalized medicine

## Abstract

*Background and objective*: IgA vasculitis (IgAV), a predominantly pediatric leukocytoclastic disease, has an unpredictable, though largely benign, evolution. The aim of this study was to retrospectively investigate any potential clinical or laboratory predictors of gastrointestinal involvement in a single-center cohort of children with IgAV. *Patients and methods*: A total of 195 children with a history of IgAV, regularly followed-up for an average period of 1 ± 2.6 years via outpatients clinics of the pediatric rheumatology unit in our University, were assessed, analyzing their clinical and laboratory variables in relationship with their disease evolution and outcome. *Results*: Univariate analysis showed that a higher neutrophil granulocyte count and lower lymphocyte count (expressed as a percentage of the total white blood cells) were significantly associated with the presence of gastrointestinal involvement at the first examination (65.2 ± 13% versus 58.8 ± 12%, *p* = 0.02, and 26.4 ± 11% versus 32.1 ± 11%, *p* = 0.02, respectively). A positive pharyngeal swab for *Streptococcus pyogenes*, a deficiency of 25-hydroxyvitamin D, a persistence of purpuric rash for more than 1 month, and purpuric lesions in the genital area were also associated with gastrointestinal involvement (*p* = 0.0001, *p* = 0.0001, *p* = 0.007 and *p* = 0.001, respectively). However, multiple logistic regressions with correction for the patients’ sex and age showed that lower 25-hydroxyvitamin D levels, persistent rash, and genital lesions were independently and significantly associated with signs of gastrointestinal involvement. We then performed a secondary analysis (both univariate and multivariate) to investigate whether vitamin D deficiency was associated with other IgAV manifestations: we found that only 25-hydroxyvitamin D deficiency remained significantly associated with gastrointestinal involvement in IgAV. *Conclusions*: Patients with IgAV and vitamin D deficiency might be more prone to developing gastrointestinal manifestations of variable severity.

## 1. Introduction

Immunoglobulin A vasculitis (IgAV), formerly known as Henoch-Schönlein purpura, is a quite typical vasculitis of small vessels, largely occurring in childhood, characterized by deposits of immune complexes containing predominantly IgA. Its hallmark is a non-thrombocytopenic purpuric rash on the buttocks and lower limbs, but the disease is also characterized by a risk of gastrointestinal, joint and renal involvement. The overall long-term prognosis of IgAV specifically depends on renal involvement, though it is largely benign [1]. Some patients can suffer from severe extra-skin manifestations, but the reasons for a worse disease evolution in a minority of children with IgAV remain undeciphered. The etiology of IgAV is not yet fully understood: it is thought that an aberrant immune response triggered by infectious or environmental agents in genetically predisposed children is the main intrinsic pathogenetic mechanism of the disease, leading to the formation of galactose-deficient IgA_1_ and related immune complexes that precipitate in the skin, gut, joints, or kidneys [2,3]. Gastrointestinal involvement is relatively common in IgAV, but can vary in severity, from mild nausea to gastrointestinal bleeding and intussusception that may require surgical intervention: in a recent study about 50% of IgAV cases had gastrointestinal involvement, and 48.9% of them were classified as severe [4]. In general terms, IgAV has an overall positive prognosis with complete recovery, highly dependent on the deposition of IgA at the renal level alone [5].

In our study we have evaluated the clinical and laboratory parameters of a single-center cohort of children with IgAV in relation to their disease evolution and, in particular, we have tried to retrospectively determine if any clinical or laboratory clues might eventually predict the occurrence of gastrointestinal manifestations in such a cohort of patients.

## 2. Patients and Methods

### 2.1. Population

We retrospectively evaluated the medical records of 201 unselected children with IgAV, who were first admitted to the outpatient care unit of pediatric rheumatology at our university hospital in the decade 2013–2023; 6 patients who were lost at minimal follow-up or with insufficient laboratory data were excluded from our analysis. All remaining 195 children underwent regular clinical examinations and urinalysis to detect possible late renal involvement or disease recurrence. All patients were Caucasians, and none of them were taking anti-seizure medications or vitamin D dietary supplementation.

### 2.2. Data Collection

We evaluated generic and numeric variables including sex, age at disease onset, presence of a typical rash not only in the lower limbs but also on the upper part of trunk, persistence of the rash for more than 1 month, presence of fever (body temperature above 38 °C) at disease onset combined with laboratory tests such as C-reactive protein (CRP), white blood cell count, serum IgA, C_3_ and 25-hydroxyvitamin D [25(OH)-vitamin D] as possible predictive factors for the occurrence of gastrointestinal, joint, or renal involvement. Serum 25(OH)-vitamin D levels were measured by automated chemiluminescence immunoassay technology. Gastrointestinal involvement was defined by the presence of abdominal clinical symptoms and bleeding signs (positive occult fecal test or even melena). The presence of arthritis and/or arthralgia, as well as genital involvement, was determined clinically. The findings of renal involvement included gross hematuria or microscopic hematuria, defined by more than 3 red blood cells per high-power field in the sediment of 10 mL of freshly centrifuged urine, and proteinuria was detected via dipstick test by presence of more than 30 mg/dL of proteins 3 times in one week or more than 150 mg of proteins in the urine collected after 24 h, and a urinary protein/creatinine ratio > 0.2. Patients undergoing treatment with corticosteroids or with non-steroidal anti-inflammatory drugs were registered, as well as those who required surgery for any gastrointestinal complications or those who required skin and/or kidney biopsy to better characterize their clinical picture. The results of a pharyngeal swab to detect *Streptococcus pyogenes* (*S. pyogenes*) infection were also recorded.

### 2.3. Inclusion Criteria

IgAV was diagnosed according to the EULAR/PRINTO/PRES criteria, which consist of the combination of a palpable purpuric rash (predominantly on the lower limbs) as a mandatory criterion with any one of the following: (a) diffused abdominal pain, (b) arthritis/arthralgia, (c) signs of kidney damage (hematuria/proteinuria), or (d) IgA deposition on a biopsy from any involved site [6]. To be eligible for our study patients had to have new-onset disease. The local Ethics Committee authorized a series of study protocols related to nutritional and environmental issues in patients with complex diseases, such as hereditary disorders or autoinflammatory and rheumatologic diseases (approval code: 2105; approval date: 5 February 2019). This study was carried out in accordance with the principles of the Declaration of Helsinki. All patients’ parents were informed about aims of this study at disease onset, and all of them signed a written consent for both the evaluation of their children’s anonymized data and unrestricted access to their medical records.

### 2.4. Exclusion Criteria

Patients were excluded if they (a) had a specific diagnosis of other vasculitides mimicking IgAV during an eventual hospitalization or at subsequent assessments; (b) had a previous history of hematuria or proteinuria; (c) were affected by any hematological disorders; (d) had a specific immunodeficiency; (e) had any exposure to severe acute respiratory syndrome coronavirus 2 infection.

### 2.5. Statistical Analysis

STATA 6.0TM software (University Station, TX, USA) was used for statistical analysis. We chose to describe continuous data in terms of mean values and standard deviations if they were normally distributed, otherwise we expressed them in terms of their medians and interquartile ranges. Dichotomous variables were expressed as totals and percentages. We conducted two distinct analyses. In the first, we divided the cohort into two groups based on the presence or absence of gastrointestinal involvement. In the second analysis, we classified the population into two groups based on their serum vitamin D level. Both gastrointestinal involvement and vitamin D levels were evaluated in the acute phase of the disease during the first visit to our outpatients care unit of pediatric rheumatology. Comparisons between groups were made using Student’s *t* test if the data were normally distributed; alternatively, they were made using the Mann–Whitney U test. Comparisons between groups described with dichotomous data were made with the chi-square test or, if at least one of the groups was smaller than 6, with Fischer’s exact test. To exclude confounding factors in the correlation between groups we performed a multivariate logistic regression analysis adjusted for sex and age, including all variables with a *p* < 0.2 in the univariate analysis. A *p* < 0.05 was considered statistically significant. We chose to avoid correcting the study for multiple testing due to its retrospective nature, which requires further perspective confirmation. We preferred notreducing the type I error for null-association to avoid increasing the risk of type II error for any notnullassociations.

## 3. General Results

### 3.1. Clinical Features of Children with IgAV

The mean age of the 195 children enrolled in our analysis was 79 ± 36 months, with an interquartile range of 12–194 months; 99 of the children (50.8%) were females. All patients were followed-up for an average period of 1 ± 2.6 years. Seven patients required skin biopsy to confirm IgAV. Skin manifestations were present in all children with IgAV, but purpura was present on more than 50% of the whole body surface in 56 cases (29%), while 38 patients (19.5%) had a rash persist for more than 1 month. No child had received vaccinations in the eight weeks before the occurrence of IgAV. Abdominal pain was experienced by 65 children (33%). Blood loss, either melena or a positive fecal occult blood test, was reported in 23 cases (12%); 4 of them (2% of the cohort) required surgery. Renal involvement was found in 29 patients (15%): proteinuria in 20 (10%) of all enrolled patients and hematuria in 24 (12%). No child had presented macrohematuria at the onset of IgAV, however, a diagnosis of Berger’s disease was established in three children (1.5% of the cohort) displaying persistent signs of renal involvement, which required a renal biopsy for confirmation. Joint manifestations were present in 123 cases (63% of the cohort) at the time of their first assessment; an edema of extremities was observed in 76 patients (39%) and genital involvement in 21 patients (11%). Fever was present in 37 children (19%) at disease onset, and headache in 9 (5%). Out of the 147 children who completed a pharyngeal swab, 35 (24%) were positive for *S. pyogenes*. The treatment modalities of IgAV were different: corticosteroids were used in 53 patients, and non-steroidal anti-inflammatory drugs in 49. The vast majority of patients had a midlongterm favorable course of the disease, but three of them displayed a severe outcome (i.e., an onset of Berger’s disease). Fifty-three children were submitted to steroid therapy at the onset of IgAV because of gastrointestinal, renal, or neurological complications. A total of 21 patients from the cohort (11%) were found to have low serum 25(OH)-vitamin D (<20 ng/mL,) suggesting a severe deficiency, whereas 154 (79%) had sufficient vitamin D levels (>30 ng/mL). To investigate whether gastrointestinal involvement was associated with other clinical or laboratory signs, we performed a statistical analysis, comparing patients with and without gastrointestinal involvement using both univariate and multivariate analysis.

### 3.2. Univariate Analysis

**Figure 1:**
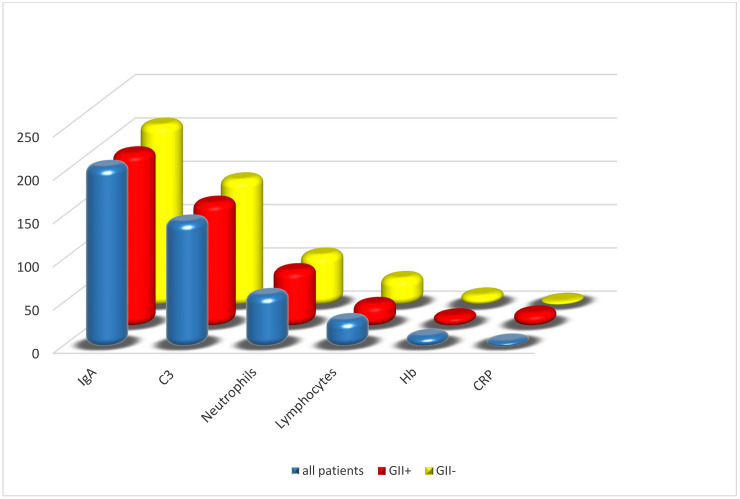
Laboratory data of patients with IgA vasculitis with or without gastrointestinal involvement. *p* = 0.02 for neutrophils and lymphocytes; *p* = ns for the other laboratory data. GII+: presence of gastrointestinal involvement; GII−: absence of gastrointestinal involvement.

We divided patients into two groups according to whether they had gastrointestinal involvement or not: the comparison of all variables revealed no statistically significant difference in terms of the patients’ sex (14 males in the group with gastrointestinal involvement versus 82 males in the other, *p* = 0.23) and the patients’ age (91 ± 37 months in the group with gastrointestinal involvement versus 78 ± 36 months in the other, *p* = 0.13). Data comparing the mean values of IgA, C_3_, CRP, hemoglobin and the percentages of both neutrophils and lymphocytes in the total count of white blood cells at the patient’s first assessment in relation to the occurrence of gastrointestinal involvement are shown in Table 1. Hemoglobin, CRP, C_3_ and IgA showed no differences between the groups; in patients with gastrointestinal involvement, the percentage of neutrophil granulocytes was significantly higher, and that of lymphocytes was significantly lower than in patients without gastrointestinal involvement (65.2 ± 13% versus 58.8 ± 12%, *p* = 0.02, and 26.4 ± 11% versus 32.1 ± 11,% *p* = 0.02, respectively). Figure 1 depicts the laboratory data found on patients with IgAV with and without gastrointestinal involvement.

Positivity for *S. pyogenes* from the throat swab, 25(OH)-vitamin D deficiency, purpuric rash persisting for more than 1 month, purpuric lesions in the genital area and the use of corticosteroids were all significantly correlated with signs of gastrointestinal involvement (see Table 2).

### 3.3. Multivariate Analysis

**Figure 2:**
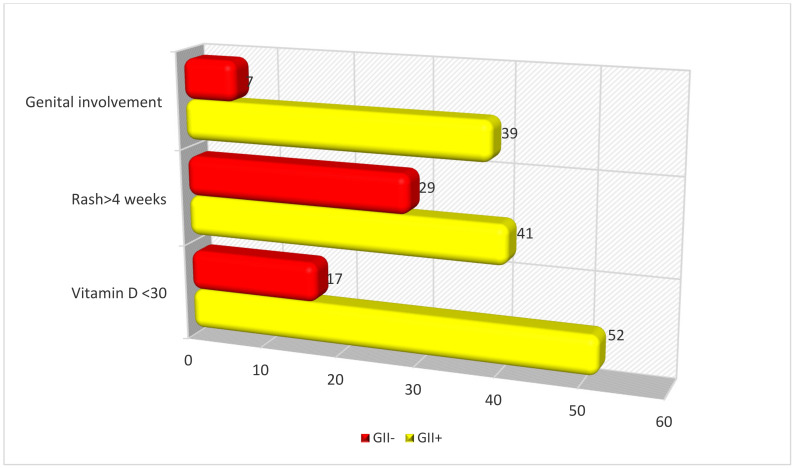
Clinical data associated with gastrointestinal involvement in the multivariate analysis of patients with IgA vasculitis. *p* = 0.003 for vitamin D < 30 ng/mL, *p* = 0.034 for rash > 4 weeks, *p* = 0.05 for genital lesions (data are expressed in percentages).

In the multivariate analysis, a serum deficiency of 25(OH)-vitamin D, persistence of the rash and genital lesions were identified as significant and independent variables correlating with gastrointestinal involvement (see Table 3). Figure 2 shows the clinical data associated with gastrointestinal involvement in the multivariate analysis of patients with IgAV.

Taking into account the association between vitamin D and gastrointestinal involvement, we also decided to conduct an additional statistical analysis, both univariate and multivariate, to explore any potential associations between vitamin D status and other systemic manifestations of IgAV.

## 4. Results of the 25-Hydroxyvitamin D Status of IgAV Patients

### 4.1. Univariate Analysis

Additionally, we divided our cohort into two groups depending on whether their vitamin D level was below 30 ng/mL or not. Table 4 shows the mean values of IgA, C_3_, CRP, hemoglobin and the percentage of both neutrophils and lymphocytes (related to the total number of white blood cells) in IgAV patients with or without 25(OH)-vitamin D deficiency, using a cut-off of 30 ng/mL. None of these variables were associated with vitamin D deficiency.

The comparison of all variables revealed no statistically significant difference between the groups with respect to sex (23 males in the group with vitamin D deficiency versus 73 males in the group with a sufficient level of vitamin D, respectively; *p* = 0.32) and age (89 ± 39 months in the first group versus 77 ± 35 months in the other, *p* = 0.09). Data comparing the laboratory tests in the groups are shown in Table 4. Among the other variables, a persistent skin rash for more than 1 month, genital involvement, the occurrence of renal manifestations/gastrointestinal signs and the use of corticosteroids were significantly associated with lower levels of 25(OH)-vitamin D (see Table 5).

### 4.2. Multivariate Analysis

During the multivariate analysis we found that vitamin D deficiency was the only significant and independent variable associated with the occurrence of gastrointestinal manifestations (see Table 6). Another dimension examined in our cohort was whether there were discrepancies in vitamin D levels depending on the month of blood collection (at disease diagnosis); examinations performed in months with increased sun exposure (from June to September) showed no statistically significant association with, or increased prevalence of, severe vitamin D deficiency compared with examinations performed in the period from October to May, in our dataset (5 out of 35, 14% versus 16 out of 160, 10%, respectively, *p* = 0.46).

## 5. Discussion

The predictive factors for gastrointestinal complications in children with IgAV have not been clearly studied, although the histopathological spectrum of endoscopic findings might help us to characterize disease severity [7]. Unlike other vasculitides occurring in childhood, such as Kawasaki disease, for which there are several recommendations in the medical literature and for which different predicting tools for disease evolution have been determined [8,9], no standardized regimen exists to manage IgAV in childhood, as large randomized controlled trials have not been conducted yet. Furthermore, there is an unmet need for reliable, validated and widely-accepted disease activity measures in children with IgAV. Renal involvement is the most important factor influencing IgAV prognosis, and there is controversy as to whether IgAV and IgAV-related nephropathy may be considered two faces of the same disease [10]. The genetic regulation of endothelial function, such as polymorphisms in genes encoding components of the renin-angiotensin system, endothelial nitric oxide synthases and intercellular adhesion molecules, could contribute to the protean expression of IgAV [11]. In addition, mutations in the *MEFV* gene associated with familial Mediterranean fever could further contribute to a predisposition to IgAV [12]. There are, at present, no measures predicting disease prognosis; however, serum interleukin-18 has been found to be significantly elevated at diagnosis in IgAV patients compared to healthy controls, and to progressively decrease as the disease turns to a positive outcome [13]. Sestan et al. have recently found that as the severity and duration of skin manifestations increase, so does the risk of developing IgAV-related nephritis and the likelihood that aggressive treatments might be required [14].

Our retrospective analysis of 195 children with IgAV has revealed that a higher percentage of neutrophils and lower percentage of lymphocytes at the first assessment were significantly associated with the occurrence of gastrointestinal involvement. In addition, a positive *S. pyogenes* pharyngeal swab, a deficiency of 25(OH)-vitamin D, a persistent purpuric rash for more than 1 month and purpuric lesions in the genital area were associated with gastrointestinal disease. However, multiple logistic regression showed that only low levels of 25(OH)-vitamin D (<30 ng/mL), persistent rash and genital lesions were independently associated with signs of gastrointestinal involvement. Further analysis (both univariate and multivariate) was performed to investigate whether vitamin D deficiency could be associated with other IgAV manifestations: our results found that only 25(OH)-vitamin D deficiency was significantly associated with the occurrence of gastrointestinal manifestations, suggesting that low levels of vitamin D may, at least one-sidedly, contribute to the development of IgAV-related gastrointestinal disease, although diet, medications and eventual comorbidities can influence vitamin D status and bias such results.

A few studies with different sample sizes had already shown that some IgAV patients might have lower serum vitamin D levels than healthy children. Zhu et al. demonstrated that leukotriene B4 was increased and 25(OH)-vitamin D decreased in children with IgAV, speculating that vitamin D deficiency might be correlated with disease severity [15]. More recently, Wang and Ye retrospectively found that 663 patients with IgAV hospitalized in a single Chinese center during a 3-year-period had reduced vitamin D levels, as did children with IgAV-related nephritis, with both a history of previous streptococcal infection and signs of gastrointestinal involvement, in comparison to 400 healthy sex/age-matched controls, suggesting that maintaining high vitamin D levels might prevent renal damage or complications in other organs [16]. In addition, Fu et al. found that recurrence rates and the incidence of renal damage in 100 children with IgAV regularly treated with vitamin D for four weeks were significantly lower than those seen in 100 children with IgAV who did not receive vitamin D after 6 months of follow-up [17].

Several autoimmune diseases tend to share a predisposition to vitamin D deficiency, which might produce an alteration of the microbiome and disrupt the integrity of the intestinal epithelial barrier [18]. It has been also suggested that vitamin D deficiency may play a role in the immune activation of patients with systemic lupus erythematosus, and have an active part in many comorbidities and even complications of this disorder [19]. Long-term treatment with vitamin D provided an enhancement of T-reg cells and production of Th2 cytokines in patients with systemic lupus erythematosus [20]. Moreover, vitamin D supplementation reduced anti-dsDNA positivity in systemic lupus erythematosus, and could possibly reduce the risk of disease recurrence, although further confirmation studies in a larger number of patients are needed [21]. Low serum concentrations of 25(OH)-vitamin D have been also found in children with Kawasaki disease, possibly contributing to the development of vascular complications in these patients [22]. Vitamin D deficiency has also been observed in children with periodic fever/aphthosis/pharyngitis/adenitis syndrome, a multifactorial non-hereditary autoinflammatory disorder of childhood, and vitamin D supplementation seemed to significantly reduce the number and overall duration of the typical inflammatory flares of this condition [23,24]. Recently, it has been reported that IgAV might have seasonal peaks, reflecting different exposure rates to infectious agents or different levels of sun exposure, which might explain an eventual subsequent hypovitaminosis D [25].

Despite the general results of our study, some limitations need to be declared as well: the retrospective nature of this analysis, the lack of a control group population, a relatively limited number of IgAV patients due to the single-centered structure of our project, a lack of data related to the dietary habits of the recruited children, and a lacking determination of IgAV prognosis in the long-term.

## 6. Conclusions

To conclude, we found that children with IgAV who are vitamin D deficient may be more likely to develop gastrointestinal manifestations of varying severity: in real-world healthcare settings, although further multi-center collaborative studies are needed to confirm and validate our findings, eventually incorporating additional potential predictors, we believe that frontline physicians might be helped in the decision making process and able to optimize the management or surveillance strategies of IgAV if it is associated with hypovitaminosis D.

## Figures and Tables

**Table 1:** Comparison of the laboratory data of patients with IgA vasculitis with or without signs of gastrointestinal involvement (GII+ and GII−, respectively).

	All Patients	GII+	GII−	*p*
IgA (mg/dL, mean ± SD)	208 ± 78	200 ± 60	209 ± 80	0.60
C_3_ (mg/dL, mean ± SD)	145 ± 39	143 ± 34	146 ± 39	0.79
CRP (mg/L, median [IQR])	7.2 (3–17)	16 (6–36)	6 (3–16)	0.24
Neutrophils (%, mean ± SD)	59.60 ± 13	65.17 ± 13	58.81 ± 12	0.02
Lymphocytes (%, mean ± SD)	31.40 ± 11	26.39 ± 11	32.12 ± 11	0.02
Hb (g/dL, mean ± SD)	12.7 ± 1	12.6 ± 1	12.7 ± 0.9	0.62

IgA: immunoglobulin A (normal reference values in relation to the patients’ age of our cohort: 40–240 mg/dL), C_3_: complement (normal reference value, 90–180 mg/dL), CRP: C-reactive protein (normal reference value: <3 mg/L), Hb: hemoglobin; IQR: interquartile range; SD: standard deviation.

**Table 2:** Univariate analysis between patients with IgA vasculitis with and without signs of gastrointestinal involvement (GII+ and GII-, respectively).

	All Patients	GII+	GII-	*p*
	N/TN (%)	N/TN (%)	N/TN (%)	
*S. pyogenes+*	35/147 (24)	0/14 (0)	35/98 (35)	0.0001
Vitamin D < 20 ng/mL	21/195 (11)	9/23 (39)	12/172 (7)	0.0001
Vitamin D > 30 ng/mL	20/19 (11)	11/23 (48)	143/172 (83)	0.0001
Rash > 50% of body	56/195 (29)	10/23 (43)	46/172 (27)	0.1
Rash > 4 weeks	38/195 (19)	9/23 (41)	29/172 (17)	0.007
Need for surgery	4/195 (2)	4/23 (17)	0/172 (0)	-
Renal involvement	29/195 (15)	4/23 (17)	25/172 (15)	0.75
Proteinuria	29/195 (15)	4/23 (17)	25/172 (15)	0.75
Hematuria	24/195 (12)	3/23 (13)	21/172 (12)	1
Berger’s disease	3/195 (2)	1/23 (4)	2/172 (1)	0.3
Edema of extremities	76/195 (39)	9/23 (39)	67/172 (39)	0.99
Joint involvement	123/195 (11)	16/23 (70)	107/172 (62)	0.49
Headache	9/195 (5)	1/23 (4)	8/172 (5)	0.35
Genital involvement	21/195 (11)	9/23 (39)	12/172 (7)	0.001
Fever	37/195 (19)	6/23 (26)	31/172 (18)	0.35
Use of corticosteroids	53/195 (56)	18/23 (78)	35/172 (20)	0.001
Use of NSAIDs	49/195 (25)	6/23 (26)	43/172 (25)	1
Positive prognosis	192/195 (98)	22/23 (96)	169/171 (99)	0.89

GII: gastrointestinal involvement; N: number; TN: total number; NSAIDs: non-steroidal anti-inflammatory drugs.

**Table 3:** Multivariate logistic regression analysis between patients with IgA vasculitis with and without signs of gastrointestinal involvement (GII+ and GII−, respectively).

	GII+	GII−	*p*	OR (95% CI)
	N/TN (%)	N/TN (%)		
Vitamin D < 30 ng/mL	12/23 (52)	29/172 (17)	0.003	5.6 (1.8–17.6)
Rash > 4 weeks	9/23 (41)	29/172 (17)	0.034	3.2 (1.1–9.2)
Genital involvement	9/23 (39)	12/172 (7)	0.05	7.1 (1.8–28.0)

GII: gastrointestinal involvement; N: number; TN: total number; OR: Odds Ratio, CI: confidence intervals.

**Table 4:** Laboratory data on patients with IgA vasculitis displaying 25(OH)-vitamin D deficiency or not.

	All Patients	Vitamin D < 30	Vitamin D > 30	*p*
IgA (mg/dL, mean ± SD)	208 ± 78	223 ± 81	204 ± 77	0.17
C_3_ (mg/dL, mean ± SD)	145 ± 39	143 ± 47	146 ± 36	0.74
CRP (mg/L, median [IQR])	7.2 (3–17)	12 (3–28)	6 (3–15)	0.24
Neutrophils (%, mean ± SD)	59.60 ± 13	61.80 ± 11	58.97 ± 13	0.18
Lymphocytes (%, mean ± SD)	31.40 ± 11	29.71 ± 11	31.91 ± 11	0.27
Hb (g/dL, mean ± SD)	12.7 ± 1	12.6 ± 1	12.7 ± 1	0.86

C_3_: complement; CRP: C-reactive protein; IQR: interquartile range; SD: standard deviation, Hb: hemoglobin.

**Table 5:** Univariate analysis between patients with IgA vasculitis with and without vitamin D deficiency.

	All Patients	Vitamin D < 30	Vitamin D > 30	*p*
	N/TN (%)	N/TN (%)	N/TN (%)	
*S. pyogenes +*	35/147 (24)	4/32 (12)	31/115 (27)	0.1
GI involvement	33/195 (17)	12/41 (29)	11/154 (7)	0.0001
Rash > 50% of body	56/195 (29)	15/41 (37)	41/154 (27)	0.21
Rash > 4 weeks	38/195 (19)	14/41 (34)	25/154 (16)	0.01
Need for surgery	4/195 (2)	4/41 (10)	0/154 (0)	0.001
Renal involvement	29/195 (15)	12/41 (29)	17/154 (11)	0.004
Proteinuria	29/195 (15)	10/41 (24)	10/154 (6)	0.001
Hematuria	24/195 (12)	10/41 (24)	14/154 (9)	0.01
Berger’s disease	3/195 (2)	3/41 (7)	0/154 (0)	0.01
Edema of extremities	76/195 (39)	18/41 (44)	58/154 (38)	0.46
Joint involvement	123/195 (11)	30/41 (73)	93/154 (60)	0.49
Headache	9/195 (5)	2/41 (5)	7/154 (5)	0.9
Genital involvement	7/154 (5)	7/154 (5)	11/154 (7)	0.01
Fever	37/195 (19)	11/41 (27)	26/154 (17)	0.1
Use of corticosteroids	53/195 (56)	29/41 (71)	24/154 (16)	0.0001
Use of NSAIDs	49/195 (25)	9/41 (22)	40/154 (26)	0.59
Positive prognosis	192/195 (99)	38/41 (93)	153/153 (100)	0.03

GI: gastrointestinal; N: number; TN: total number; NSAIDs: non-steroidal anti-inflammatory drugs.

**Table 6:** Multivariate logistic regression analysis between patients with IgA vasculitis with and without vitamin D deficiency.

	Vitamin D < 30	Vitamin D > 30	*p*	OR (95% CI)
	N/TN (%)	N/TN (%)		
GI involvement	12/41 (29)	11/154 (7)	0.02	1.7 (1.24–8.89)

GI: gastrointestinal; N: number; TN: total number; OR: Odds Ratio, CI: confidence intervals.

## Data Availability

No new data were created or analyzed in this study. Data sharing is not applicable to this article.
